# Early and Multidisciplinary Management of Chronic Noncancer Pain and Conceptualization of Central Sensitization in Spain: Delphi CADOR Study

**DOI:** 10.1155/prm/1295794

**Published:** 2026-10-04

**Authors:** Carlos Tornero, Pedro Juan Ibor Vidal, Carlos Ibor Miguel, Manuel Maestre Moreno, Luis Javier Roca Ruiz, Maria Ruart, Angela Magrone Massari

**Affiliations:** ^1^ Chief of the Pain Unit Department of Anesthesiology, Reanimation and Pain Therapeutics (SARTD) University Clinical Hospital of Valencia Valencia Spain; ^2^ Department of Surgery University of Valencia Valencia Spain uv.es; ^3^ Vice President of the Spanish Multidisciplinary Pain Society (SEMDOR) Madrid Spain; ^4^ Physician at the Department of Rehabilitation and Physical Medicine University Clinical Hospital of Valencia Valencia Spain; ^5^ Faculty of Medicine Catholic University of Valencia Valencia Spain ucv.es; ^6^ Department of Family and Community Medicine in Córdoba La Carlota Health Center Córdoba Spain; ^7^ Chief of the Department of Orthopedic Surgery and Traumatology University Hospital Virgen Macarena of Sevilla Sevilla Spain; ^8^ Department of Surgery, Traumatology and Orthopedics University of Sevilla Sevilla Spain us.es; ^9^ Adelphi Targis S.L Barcelona Spain; ^10^ Medical Department Ferrer Internacional, S.A. Barcelona Spain

**Keywords:** CNCP, derivation, diagnoses, follow-up, interdisciplinary, treatment

## Abstract

**Background:**

The CADOR study aimed to obtain consensus on the diagnosis, management, and follow‐up of patients with chronic noncancer pain (CNCP) in Spain, including beliefs about central sensitization (CS), and addressing multidisciplinary care and referral processes.

**Methods:**

A Delphi consensus of two successive rounds (July 2023–November 2023) among 100 panelists working in Primary Care (PC), Traumatology, Rehabilitation, and Pain Units (Anesthesiology) was performed. Consensus was defined as ≥ 70% of panelists agreeing or disagreeing with a statement. The questionnaire included 30 questions with some Likert‐type scale items systematized as follows: 1 to 3 (*disagreement*), 4 to 6 (*neutral*), and 7 to 9 (*agreement*). A scientific committee (SC) of five experts in the field validated the questionnaire and its results.

**Results:**

Panelists considered pain as an entity and underlined the need to include it in medical records. CNCP should be addressed holistically and managed by a multidisciplinary team. Experts agreed to specifically investigate the presence of pain in patients with mood disorders and among the elderly. Moreover, the panelists considered it important to highlight the role of CS in CNCP evolution, a concept not well established in real clinical practice. Panelists emphasized the need to enhance knowledge to prevent the chronification of pain, address symptoms potentially associated with CS, and to improve patients’ prognosis. Finally, panelists highlighted the need for ongoing training initiatives for clinicians and patients, promoting awareness, with a particular focus on the patients’ needs.

**Conclusion:**

This study underscores the importance of early detection, personalized treatment, and structured follow‐up for the correct therapeutic approach of CNCP in the Spanish setting. These recommendations may also help to anticipate symptoms potentially associated with CS by improving conceptual understanding in CNCP patients. Finally, respondents also highlighted the relevance of multidisciplinary care and referral processes in patients with CNCP.

## 1. Introduction

Pain is defined as “*an unpleasant sensory and emotional experience associated with actual or potential tissue damage or described in terms of such damage”* [1]. When pain persists for more than 3 months, beyond the expected period of healing, presents with moderate or high intensity, and is not associated with an oncological process, it is defined as chronic noncancer pain (CNCP) [2, 3]. With a global prevalence between 12% and 42% [4], CNCP affects approximately 17% of adults in Spain, with a higher incidence in women and the elderly [5]. In Spain, the main reported diagnosis associated with CNCP includes fibromyalgia/chronic fatigue (40%), headache (24%), and musculoskeletal problems, such as low back pain (24%) and osteoarthritis (23%) [6].

CNCP affects patients’ functioning (loss of autonomy, difficulties in daily activities) and psychological health (anxiety, depression), leading to an impairment in the self‐perceived quality of life (QoL), and significant social, occupational, and family consequences. [7]. Although nonfatal, CNCP is a major cause of disability worldwide as measured by years lived with disability (YLDs) and is associated with substantial healthcare costs [8]. The economic burden is substantial, with estimated annual costs of €300 billion in Europe and €16 billion in Spain [9, 10]. Given its high prevalence and significant personal and socioeconomic impact, CNCP is a major public health concern.

CNCP is a multifactorial entity with a pathophysiological origin but is also influenced by psychological and social factors. According to its pathophysiological origin, pain may be classified as nociceptive (associated with real or potential tissue damage), neuropathic (caused by nerve damage), or mixed (a mixture of the aforementioned) [11, 12]. Recently, nociplastic pain has been defined as a consequence of augmented central nervous system (CNS) nociceptive processing and altered pain modulation. However, it is still not well understood [13, 14]. In addition, chronic pain has frequently been associated with central sensitization (CS), which is an increased responsiveness of central nociceptive neurons [15]. Some mechanisms are proalgesic, resulting in generalized pain amplification within the CNS, whereas others belong to endogenous pain inhibitory pathways [16]. Therefore, CS may result in either a decrease or an increase in the pain threshold in response to nociceptive stimuli [17–20]. With this caveat in mind, it is of utmost importance to correctly identify the type of pain to design an effective management strategy, since neuropathic and mixed pain is more difficult to treat than pure nociceptive [21–23].

Given its multifactorial etiology and its subjective nature, CNCP is a challenging condition to manage. It commonly requires a multimodal and multidisciplinary approach including Primary Care (PC), specialists, and Pain Units [24]. However, CNCP is largely underdiagnosed and undertreated worldwide. In fact, median time to diagnosis is 2.2 years, and up to 13% of patients do not receive a proper diagnosis in Spain [6, 10]. In addition, half of patients with chronic pain report being dissatisfied with the time it took to get adequate management of their pain [6]. Since no curative therapies are currently available, patients should be informed of this limitation. Therapeutic goals should be realistic and aimed at pain reduction rather than curing it, to maximize patients’ QoL. CNCP is commonly managed with nonpharmacological (psychoeducation and physical therapy) and pharmacological approaches, according to the particular patient’s needs. Pharmacological treatment includes pain relievers, opioids, antidepressants, and antiseizure drugs, or drug combinations in routine clinical practice [25].

Although several management recommendations on CNCP have been published, they are mainly focused on pharmacological treatment and do not address beliefs or conceptualization of CS in depth, nor the detection and management of CNCP. This study aimed to gather expert opinions on the management of patients with CNCP in the Spanish context, as well as on the importance of early diagnosis and optimal treatment, in order to avoid the chronification of pain and anticipate the appearance of potentially associated CS symptoms. Special emphasis was also placed on assessing the relevance of multidisciplinary care and the referral pathways for patients with CNCP and associated CS. In Spain, existing national guidance on chronic pain management remains largely focused on nociceptive mechanisms and pharmacological treatment, with limited integration of CS concepts or CS‐specific assessment tools in routine pathways, particularly in PC [24, 26, 27, 29]. Consequently, clinicians often rely on heterogeneous, nonstandardized approaches to recognizing CS‐related symptoms, and there are no widely adopted protocols for early CS identification or referral. This context makes it especially relevant to explore how Spanish physicians currently conceptualize CS and how this understanding can be better aligned with national clinical practice.

## 2. Material and Methods

### 2.1. Study Design

A traditional Delphi method was used to obtain a consensus among a panel of experts regarding the diagnosis and management of patients with CNCP in Spain. The process consisted of two rounds of anonymous responses without direct interaction among panelists. The Delphi process followed 5 phases: (1) conceptualization (including the creation of the scientific committee [SC] and project definition); (2) literature review and questionnaire design; (3) recruitment of panelists; (4) administration of the two Delphi rounds; and (5) data collection and statistical analysis.

The SC included 5 experts in CNCP management in Spain. Their responsibilities included conceptualizing and validating the questionnaire, rewording items before the second round (if necessary), and discussing and validating the final Delphi results.

### 2.2. Design of the Delphi Questionnaire

The questionnaire consisted of 30 questions, including single‐choice, multiple‐choice, and Likert‐type questions. The questions contained both descriptive items (11 items) and agreement statements (168 items). The statements were distributed in 4 main areas: CNCP definition (4 items of agreement); CNCP detection and diagnosis (62 items of agreement + 10 descriptive items); CNCP treatment (82 items of agreement + 1 descriptive item); and CNCP follow‐up (20 items of agreement) (Supporting Tables 1–4). Additionally, a small number of nonconsensus questions were included to gain deeper insight into routine clinical practice. These questions were formatted as yes/no responses and were not considered part of the consensus process (Supporting material).

The relatively high number of items (168 agreement statements) was intentionally chosen to cover, in a granular manner, the full spectrum of CNCP definition, detection, treatment, follow‐up, and CS‐related issues. To preserve response validity in spite of potential fatigue and central tendency bias, items were grouped by domain, the online platform allowed pausing and resuming the survey, and the SC reviewed response distributions between rounds to identify patterns suggestive of nondiscriminant answering. The Delphi questionnaire and all critical comments were originally written in Spanish and were translated into English for the purposes of this manuscript.

### 2.3. Panel Selection and Survey Administration

Panelists from different regions of Spain working on PC (*n* = 48), Traumatology (*n* = 20), Rehabilitation (*n* = 20), and Pain Units (Anesthesiology, *n* = 12) were invited to participate. To be eligible, panelists had to manage at least 15% of patients reporting pain as a reason for consultation and have > 3 years of experience managing patients with CNCP.

The Delphi questionnaire was administered in two rounds through an online platform that ensured data anonymity and confidentiality. All statements not reaching consensus after the first round (< 70%) were presented again in the second round. If necessary, statements that reached the “undetermined” level of consensus in the first round were slightly reworded before the second round to improve understanding with the agreement of the whole SC (indicated in Supporting Table 2). The first round was conducted from July 10^th^, 2023, to September 26^th^, 2023, and the second round from November 6^th^, 2023, to December 5^th^, 2023.

### 2.4. Data Analysis

Likert‐type items were rated on a 9‐point scoring scale: 1 to 3 (*disagreement*), 4 to 6 (*neutral*), and 7 to 9 (*agreement*). The percentage of panelists scoring in each three‐point region (1–3, 4–6, and 7–9) was calculated. When ≥ 70% of panelists ranked a statement between 1 and 3, the item was considered to have reached “consensus in disagreement.” When ≥ 70% of panelists ranked a statement between 7 and 9, the item was considered to have reached “consensus in agreement.” Otherwise, the level of consensus for that item was considered “undetermined.” For items with binary response options (Yes/No), the degree of agreement is shown as a percentage of respondents. Nominal variables were described using numbers and percentages. Continuous variables were described using mean and standard deviation (SD). The level of statistical significance was set at 0.05 (two‐sided).

## 3. Results

A total of 100 physicians with expertise in CNCP from across Spain were included in the panel, all of whom completed both rounds of the Delphi survey. Demographic characteristics and medical specialties of participants can be found in Table 1. The panelists were predominantly male (69%), worked mainly in public healthcare centers (80.7%), included a high proportion of PC specialists (48.0%), and reported extensive expertise (> 15 years) in CNCP management (68%).

**TABLE 1:** Characteristics of included panelists.

	*N* total = 100
Gender, *n* (%)	
Male	69 (69%)
Female	31 (31%)
Age (in years), mean (± SD)	50.6 (± 9.3)
Medical specialty, *n* (%)	
Primary Care	48 (48%)
Rehabilitation	20 (20%)
Traumatology	20 (20%)
Pain Unit (Anesthesiology)	12 (12%)
Professional activity, %	
Public	80.7%
Private	19.3%
Years of experience in managing patients with CNCP, *n* (%)	
> 15 years	68 (68%)
6–15 years	27 (27%)
< 6 years	5 (5%)
Geographical distribution, *n* (%)	
Andalusia	20 (20%)
Aragon	2 (2%)
Asturias	3 (3%)
Basque Country	4 (4%)
Canary Islands	6 (6%)
Castilla‐La Mancha	6 (6%)
Castilla y León	6 (6%)
Catalonia	11 (11%)
Community of Madrid	8 (8%)
Extremadura	3 (3%)
Galicia	11 (11%)
La Rioja	1 (1%)
Region of Murcia	3 (3%)
Valencian Community	12 (12%)

*Note: n*: number of participants.

Abbreviations: CNCP, chronic noncancer pain; SD, standard deviation.

At the end of the second round of the Delphi process, consensus was reached on 153 out of 168 items (91.1%), including 134 in “agreement” and 19 in “disagreement.” The remaining 15 items (8.9%) did not reach consensus and were classified as “undetermined.”

### 3.1. CNCP Definition

All items related to the definition of CNCP reached a high level of consensus. Panelists agreed on defining CNCP as pain present for > 3 months, continuously or intermittently, more than 5 days per week (91%) and characterized by impaired functional capacity (86%). Additionally, 84% of panelists agreed that CNCP should be considered a clinical entity. Finally, 80% of panelists agreed that CNCP is typically characterized by moderate‐to‐high intensity on the Visual Analogue Scale (VAS) (Supporting Table 1).

### 3.2. Detection and Diagnosis of CNCP

59% of panelists stated that they are unable to record CNCP in clinical records, and 80% supported including CNCP as a disease entity in patients’ medical records (Figure 1; Supporting Table 2).

**FIGURE 1:**
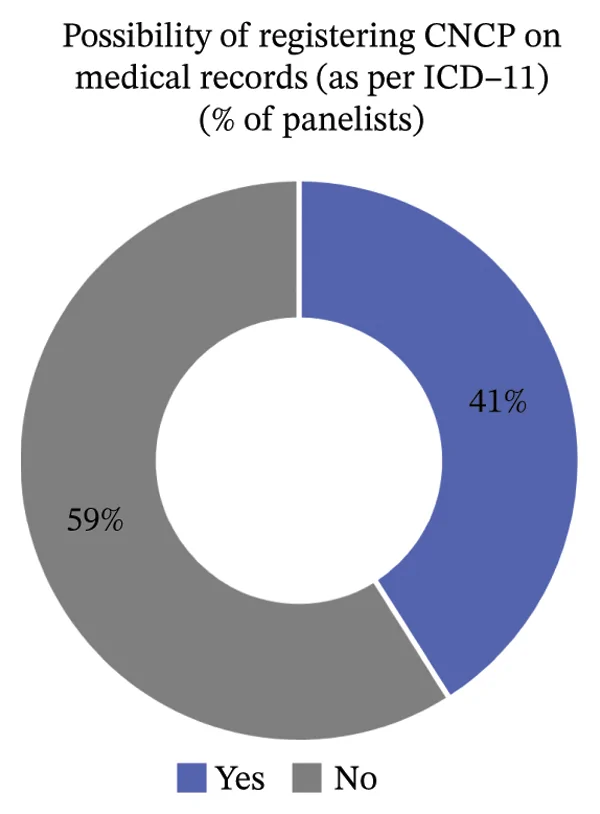
CNCP on clinical records: registration option as a clinical entity. Abbreviations: CNCP: chronic noncancer pain; ICD‐11: International Classification of Diseases 11th revision.

Regarding CNCP detection, most panelists considered it important to screen for pain in specific patient profiles (89%). This need was highlighted particularly for patients with mood disorders, such as anxiety, depression, or catastrophizing tendencies (74.2%), and for patients over 70 years of age (73%) (Figure 2; Supporting Table 2).

**FIGURE 2:**
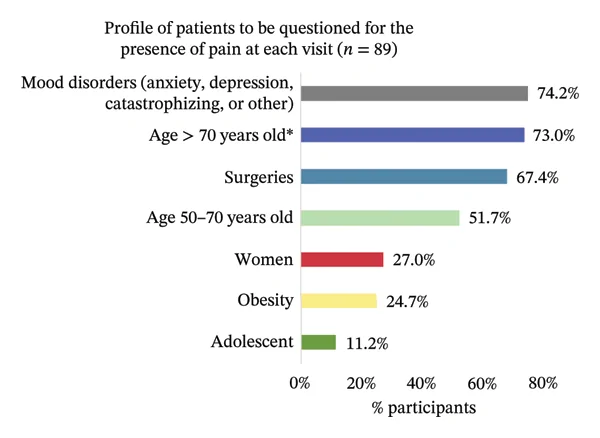
Investigation of the presence of pain in specific patient profiles. Note: ^∗^In the first round, this statement was formulated as follows: “Age > 50 years.” After the first round, the scientific committee decided to modify it to improve its comprehensibility in the second round. Abbreviation: *n*: number of participants.

For diagnosis, the vast majority of panelists agreed that it is essential to assess the impact of pain on QoL and functional capacity (95%). They also agreed that CNCP should be detected/diagnosed by the healthcare professional who first sees the patient (74%). Therefore, PC should play an essential role in CNCP detection/diagnosis as it represents the first point of entry for patients into the healthcare system (89%). They also considered the use of validated pain assessment tools essential for diagnosis (82%) (Supporting Table 2).

Regarding the initial characterization, panelists reached a high level of consensus on all statements (Supporting Table 2). As diagnostic criteria, most panelists (96%) agreed that CNCP affects QoL in physical, psychological, and social domains; it lasts ≥ 3 months, with intermittent pain ≥ 5 days a week (94%), and it persists beyond the expected time for tissue healing or resolution of the underlying disease (81%). No consensus was reached on whether the onset of pain is progressive, even after the item was reworded before the second round. There was also no consensus on whether pain intensity ≥ 3 on the Verbal Numerical Scale (VNS) should be considered a diagnostic criterion (Figure 3(a), Supporting Table 2).

**FIGURE 3:**
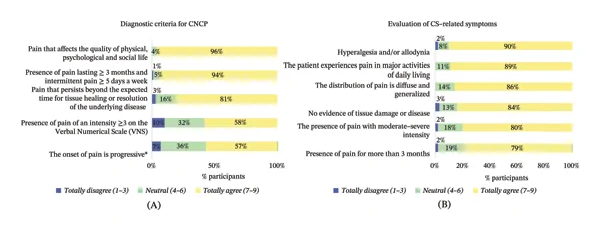
Diagnostic criteria. (A) Diagnostic criteria for CNCP; (B) evaluation of CS‐related symptoms. Note (A): ^∗^In the first round, this statement was formulated as follows: “The onset of pain is definite.” After the first round, the scientific committee decided to modify it to improve its understanding in the second round. Abbreviations (A): CNCP: chronic noncancer pain; (B) CS: central sensitization.

With respect to the pathophysiological cause of pain, most panelists (83%) considered that CNCP can be diagnosed even if its pathophysiological origin is unknown. Moreover, they agreed that knowledge of the pathophysiology of pain is essential for a correct CNCP diagnosis (76%), and pain classification should be carried out by PC (72%) (Supporting Table 2).

Considering the general knowledge of CS, a significant percentage of experts reported limited awareness of its broader implications in pain pathophysiology (69%) (Figure 4(a)). However, most panelists agreed with the conceptual definition of CS as an amplification of nociceptive signaling within the CNS resulting in pain hypersensitivity (94%) and recognized that symptoms potentially associated with CS should be considered a medical condition with a negative impact on patients’ QoL (94%). Moreover, resources and sufficient time should be provided to explain to patients with CS‐related symptoms what their medical condition entails and the available therapeutic options (96%). Panelists also agreed that CS should be considered in all patients, regardless of the type of pain (71%), as acute pain may trigger CS, lowering the pain threshold and increasing the response to noxious stimuli (71%) (Figure 4(b); Supporting Table 2). No consensus was reached on whether the detection and possible interpretation of CS symptoms should be made by PC, although panelists agreed that the identification of symptoms potentially associated with CS should be carried out by PC with support from other clinical areas such as Rehabilitation, Traumatology, Rheumatology, and/or the Pain Unit with a high level of consensus (93%) (Figure 4(b); Supporting Table 2).

**FIGURE 4:**
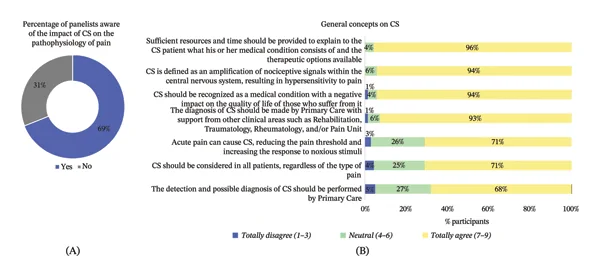
Knowledge about CS and its impact on CNCP. (A) Percentage of panelists aware of the impact of CS on the pathophysiology of pain; (B) general concepts on CS. Abbreviations: CNCP: chronic noncancer pain; CS: central sensitization.

Overall, panelists reported not having enough time for the evaluation and suspicion of symptoms of CS (81%), and cases involving complex patients and those with symptoms of CS are treated by other specialties (74%). Importantly, only 12% of panelists reported using tools for CS evaluation in CNCP patients, with the lack of consultation time being the main reason for not using these tools. Finally, panelists agreed on not having protocols or strategies for the confirmation and/or management of CS being available at their usual care center (74%). However, no consensus was reached on whether there are enough pain training courses offered (53%), even after the question was reworded before the second round (Supporting Table 2).

Regarding the criteria for assessing CS symptoms, panelists agreed on considering: hyperalgesia and/or allodynia (90%), pain experienced by the patient in major activities of daily living (89%), diffused and generalized pain (86%), no evidence of tissue damage or disease (84%), and moderate‐to‐severe pain (80%) lasting more than 3 months (79%) (Figure 3(b); Supporting Table 2).

Regarding referral criteria at the time of diagnosis, most panelists (90%) agreed that patients with CNCP and joint range limitations should be referred to a Rehabilitation specialist. In addition, patients with uncontrolled CNCP of musculoskeletal origin and/or a suspected surgical indication should be referred to a Traumatology specialist (89%); and vulnerable patients, such as pregnant women or patients on multiple medications, to Rehabilitation or Pain Units (79%). Panelists mostly agreed on referring patients with pain and a VAS score ≥ 8 (81%), suspected presence of CS (78%), and patients with painful diabetic neuropathy, postherpetic neuralgia, cervical and lumbar radiculopathy, or entrapment neuropathies (77%) (Figure 5; Supporting Table 2).

**FIGURE 5:**
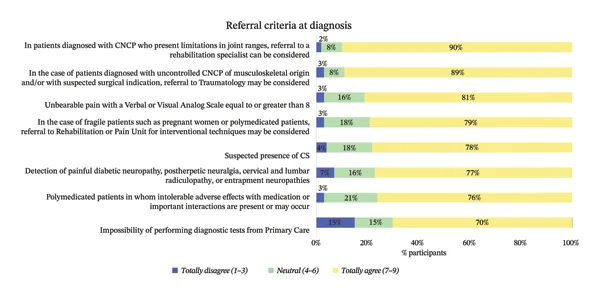
Referral criteria at the time of diagnosis. Abbreviations: CNCP: chronic noncancer pain; CS: central sensitization.

### 3.3. CNCP Treatment

The vast majority of participants agreed that the main therapeutic goal should be to reduce pain intensity and its functional consequences (Supporting Table 3). For general management, the importance of improving patient–physician communication to avoid creating false expectations of treatment efficacy (94%) was also highlighted.

Irrespective of the pathophysiological cause, special treatment and care are required early on in chronic pain (94%), and it is important to assess treatment adherence (93%). Moreover, nonpharmacological alternatives (92%) as well as disease education (90%) should be offered to patients to improve adherence. Minimally invasive procedures should be considered from the outset, even for mild to moderate pain intensity, as they can interrupt the course of pain and prevent its chronification and CS phenomena (88%), because inadequate treatment leads to pain chronification (88%). Some patient profiles, such as those with locomotor system pathology, could benefit from physical therapy or other modalities (87%) (Supporting Table 3).

Respondents (82%) reported a high concern about CS in the treatment and prognosis of patients with CNCP (Figure 6(a); Supporting Table 3). Panelists agreed that several medical specialties (Rehabilitation, Traumatology, PC, Rheumatology, and/or Pain Units) should be involved in the management of patients with symptoms potentially associated with CS (94%). Complex cases should be managed by PC and other clinical areas (93%), and mixed CNCP should be treated by a multidisciplinary team (92%) (Figure 6(b); Supporting Table 3).

**FIGURE 6:**
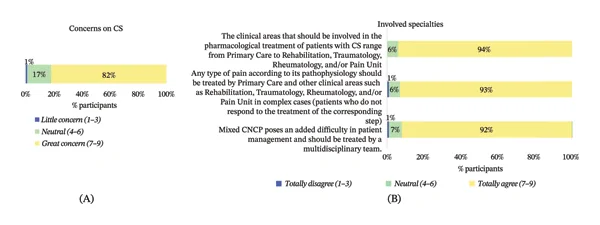
Concerns on CS of associated symptoms on treatment and prognosis in patients with CNCP. (A) Concerns on CS; (B) involved specialties.

The use of antipyretic analgesics and nonsteroidal anti‐inflammatory drugs (NSAIDs) was also considered in several patient profiles, including, but not limited to, patients with mild pain (91%), as a first step (89%), and in patients < 18 years old (86%). Conversely, panelists agreed not to use these drugs in patients with comorbidities (88%), neuropathic pain (86%), as a third step (84%), and as a treatment of CS‐related symptoms (75%). No consensus was reached on the use of antipyretic analgesics and NSAIDs in patients without pain control, in elderly patients, and in patients receiving chronic opioid treatment (Table 2, Supporting Table 3).

**TABLE 2:** Recommended and nonrecommended^∗^ pharmacological treatment per patient profiles.

	Pharmacological treatments			
Patient profiles	Antipyretic analgesics and NSAIDs	Weak opioids	Strong opioids	Antidepressants, antiseizure, or antiepileptic drugs
Patient with mild pain	Recommended	Not recommended	Not recommended	Not recommended
Patient with moderate pain	Recommended	Recommended	Not recommended	Recommended
Patient with severe pain	Not recommended	Recommended	Recommended	Recommended
As a first step	Recommended	Not recommended	Not recommended	Not recommended
As a second step	Not recommended	Recommended	Not recommended	Recommended
As a third step	Not recommended	Undetermined	Recommended	Recommended
Young patients < 18 years old	Recommended	Not recommended	Not recommended	Not recommended
Elderly patients	Undetermined	Recommended	Undetermined	Recommended
Patient with nociceptive pain	Recommended	Recommended	Recommended	Recommended
Patient with neuropathic pain	Not recommended	Recommended	Recommended	Recommended
As a treatment for CS	Not recommended	Recommended	Recommended	Recommended
Patients with suboptimal pain control/no pain control	Undetermined	Recommended	Recommended	Recommended
Patients with comorbidities^†^	Not recommended	Recommended	Recommended	Recommended
Polymedicated patients	Not recommended	Recommended	Recommended	Recommended
Patient in chronic treatment with opioids	Undetermined	Undetermined	Recommended	Recommended

*Note:* ^∗^It is indicated as “recommended” when ≥ 70% of panelists agreed on the use of the pharmacological group for each specific patient profile. Conversely, it is indicated as “not recommended” when ≥ 70% of panelists disagreed. It is indicated as “undetermined” when no consensus was reached on the use of that pharmacological treatment for that patient profile.

Abbreviations: CS: central sensitization; NSAIDs: nonsteroidal anti‐inflammatory drugs.

^†^Renal failure, liver failure, cardiac or respiratory failure, arterial hypertension, cerebral and cerebrovascular diseases, digestive diseases (e.g., peptic ulcers), obesity, anxiety.

The use of weak opioids was considered indicated in patients with nociceptive pain (90%), with comorbidities (89%), in elderly patients (89%), and as a treatment of CS‐related symptoms (83%). On the contrary, panelists disagreed with its use for mild pain (90%), as a first step (85%), and in patients < 18 years old (72%). No agreement was reached on the use of these pharmacological groups as a third‐step treatment, and in patients on chronic opioid treatment (Table 2; Supporting Table 3).

Regarding the use of strong opioids, panelists recommended them for severe pain (96%), as a third step (95%), in patients without pain control (91%), and as a treatment for CS‐related symptoms (80%). The use of strong opioids was discouraged for mild (95%) or moderate pain (79%), and as a first (93%) and second step (82%). Regarding the use of strong opioids in the elderly, no agreement was reached (Table 2; Supporting Table 3).

As for the use of antidepressants, antiseizure, or antiepileptic drugs, experts agreed to use them for neuropathic pain (97%), as a treatment for CS‐related symptoms (93%), and in patients without pain control (92%). However, the panel disagreed with using these drugs for mild pain (90%), in patients < 18 years old (84%), and as a first step (83%) (Table 2; Supporting Table 3).

Regarding referral criteria at the time of treatment, most panelists agreed to refer nonresponding patients with severe pain (90%) or those receiving very high doses of oral opioids (90%). They also agreed to refer patients with mobility restrictions (86%) and patients with CNCP of osteoarticular origin on physical treatment without any clinical improvement (81%) to the Rehabilitation Service. There was broad agreement for the referral of individuals with allergy to a wide spectrum of drugs, drug intolerance, or adverse effects (83%); patients with an established chronic degenerative disease, presenting with acute clinical manifestations different from the underlying disease for < 3 months old, that limits basic autonomy (81%); patients with a concomitant condition with contraindication to certain pharmacological pain treatments (81%); and patients incapable of receiving oral analgesic drugs, or for whom transdermal administration is not recommended (75%) (Figure 7; Supporting Table 3).

**FIGURE 7:**
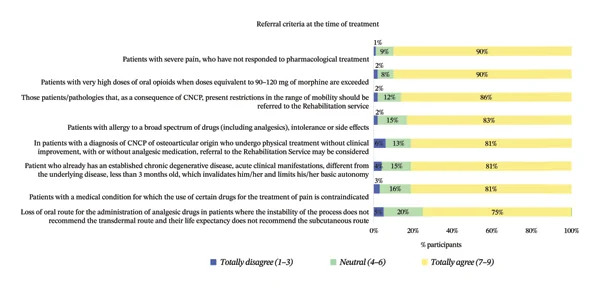
Referral criteria at the time of treatment. Abbreviation: CNCP: chronic noncancer pain.

Panelists agreed to develop a personalized follow‐up (94%), which should be even closer in patients with comorbidities (94%), and in patients receiving opioid treatment (90%). This follow‐up should be performed by PC (90%) and nursing staff (84%), and in complex cases, by other clinical areas (89%). Additionally, patients should be referred to a patient education program (78%) (Supporting Table 4). Referral criteria at follow‐up included patients with: symptoms associated with CS whose pharmacological treatment is ineffective (93%), either uncontrolled neuropathic pain or those on the third step of treatment (92%), chronic pain refractory to an adequate therapeutic regimen (91%), failure to achieve functional goals after 6 months of treatment (90%), unmanageable misuse of strong opioids (86%), compromised psychological status (80%), intolerable side effects from oral opioids (74%), and in cases of rapid tolerance to oral opioids (71%) (Figure 8; Supporting Table 4).

**FIGURE 8:**
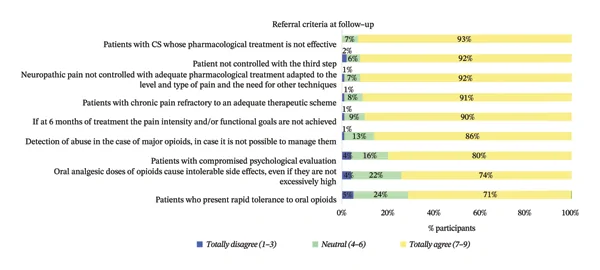
Referral criteria at follow‐up. Abbreviation: CS: central sensitization.

## 4. Discussion

To our knowledge, this is the first study that addresses the detection, diagnosis, and management of patients with CNCP in Spain, while also addressing the conceptualization of CS. Our findings highlight that CS remains a poorly established concept in routine clinical practice, and emphasize the urgent need to improve healthcare professionals’ understanding of CS to support early intervention and prevent pain chronification.

Overall, the majority of statements reached consensus either in the first or second round of the Delphi survey, suggesting a homogeneous approach to CNCP diagnosis and management among experts. Real‐world implementation of recommendations presented in this work could help to improve the detection, management, and follow‐up of CNCP, and this might result in personal benefits, QoL improvement, and socioeconomic gain and ultimately optimize the suspicion of the symptoms associated with CS. Considering that CNCP prevalence increases with age, and estimating that in 2037, the population over 65 years old will account for 26.0% of the total Spanish population [29], and CNCP prevalence will increase substantially in the future in our country. Therefore, it is time to take action so that CNCP can be appropriately managed and prioritized in public health policies.

Although there was broad agreement on CNCP definition, the classification of CNCP as a disease in its own right remained controversial, requiring two rounds of voting. This reflects ongoing debate in the scientific community about whether pain is an independent disease entity or merely a symptom of underlying conditions. The lack of a universally accepted definition has contributed to this uncertainty. Nevertheless, in 2015, the IASP Classification of Chronic Pain for the ICD‐11 clearly distinguished between chronic primary pain (e.g., in fibromyalgia or migraine) and secondary pain syndromes [2, 30]. Progressively, more publications and medical associations have accepted pain as an entity in itself [2, 31–33], although further dissemination of pain definition should be endorsed for its broader acceptance. In either case, chronic pain is a long‐term condition that should be precisely managed. For this reason, it must be properly identified and recorded. However, a slight majority of experts (59%) stated the impossibility of registering CNCP in clinical records, hampering appropriate management and making it necessary to implement new protocols in this line.

Panelists believed it was critical to assess CNCP in specific patient profiles, such as those with mood disorders. Indeed, it has been reported that 30%–40% of pain sufferers have concomitant anxiety, depression, or catastrophizing, being more prevalent in women [7]. In addition, the psychological impact of pain should not be overlooked, because mood disorders may cause fear of performing activities that may worsen pain, generating a vicious circle of fear and avoidance [7]. In addition to considering pain in a general sense for any surgery, attention should also be paid to the psychosocial state before surgery since one of the factors most associated with CNCP development is the presurgical mood, because many chronic pain conditions have been proposed to entail a relevant cognitive–behavioral component [34, 35].

According to this consensus, elderly patients (> 70 years old) were also identified as a priority group for CNCP assessment. This is, on the one hand, because prevalence increases with age; and on the other hand, being > 65 years old is a factor associated with a higher pain‐related impairment [36]. The presence of chronic pain after surgery is frequently reported with prevalences ranging from 10% to 50%, but panelists did not reach a consensus on investigating the presence of pain in this specific patient profile [37–39]. While much effort has been put into postoperative pain management, appropriate care has not yet been achieved, and the prevalence remains unchanged. Clinical practice is quite focused on pain relief arising from major surgery, which is considered more painful than minor surgery, and perhaps optimal analgesic treatment is not provided [40]. Whatever the case, pain should be considered across the entire surgical spectrum due to its high prevalence and consequences [37–39].

A wide consensus on diagnostic criteria for CNCP, considering its frequency (≥ 3 months and intermittent pain during ≥ 5 days a week) and personal impact (physical, psychological, and social), was reached in the present study. These criteria are in line with previously published consensus and guidelines [24, 26–28, 39, 41–47]. In contrast, no consensus was reached on whether the progressive onset of pain might be considered a diagnostic criterion. This may be because mild‐intensity pain could also be classified as CNCP. Finally, no consensus was reached regarding the presence of pain of an intensity ≥ 3 on the VNS as a diagnostic criterion. However, VNS is a validated tool that positively correlates with other pain measures and shows sensitivity to pain treatments [48].

Early diagnosis is crucial for improving prognosis, limiting functional decline, and preserving QoL. Patients with pain waiting for 6 months to receive a treatment experience a significant decline in QoL and psychological well‐being [49]. Given that PC is the first point of entry for patients into the healthcare system, it ought to play an essential role in the detection/diagnosis of CNCP. For proper CNCP detection and diagnosis, it is important to use validated pain assessment scales such as the VAS [50]. However, up to 10% of panelists reported not using these scales. This may be partially explained by the limited time that can be dedicated to patients during the consultation. Moreover, it highlights the relevance of including other specialties such as Pharmacy and Nursing in multidisciplinary management [51–53]. Indeed, panelists agreed on the importance of nursing in patients′ follow‐up and in outcome assessment using scales.

A noteworthy finding is that many experts were unfamiliar with the implications of CS in chronic pain pathophysiology. Notably, this observation should be interpreted alongside the finding that 94% of panelists agreed with the conceptual definition of CS. Therefore, the results do not necessarily indicate a lack of recognition of the concept itself, but rather a limited familiarity with its broader pathophysiological relevance and clinical implications. This discrepancy suggests that, although the concept of CS is generally recognized, its practical interpretation and application in routine clinical practice may still be evolving. This may be due to the relative novelty of the concept and limited integration into general clinical training, particularly for PC physicians [54]. This result might also be explained by the fact that almost half of the participants were PC physicians but not pain specialists. There was no consensus on whether the detection of symptoms potentially associated with CS should be performed by PC alone, but there was agreement that certain pain profiles may be associated with CS (nociception) by PC physicians, with support from other clinical areas, such as Rehabilitation, Traumatology, Rheumatology, and/or the Pain Unit. These results could also be explained by the fact that the assessment of symptoms potentially associated with CS would be made by the first specialist to see the patient.

Interestingly, a low percentage of panelists recognized the use of “CS detection tools.” CS is not yet widely established within the scientific community [55]. In fact, CS was originally proposed as a clinical term to explain the underlying mechanism of some pain clinical conditions. The authors report that clinical pain specialists do not know what CS is and how relevant it is in clinical practice, though they claim that it is important. This could be explained by the miscommunication generated between the fundamental pain neuroscience substantiating CS, and its translation to the clinics, and the diagnosis/treatment of pain, offered by the current bibliographic landscape about CS in the clinical field.

Some experts consider that CS could give a neurobiological explanation for chronic pain [56]. Contrarily, others adopt a more conservative perspective on CS as a phenomenon correlated with chronic pain, avoiding interference from CS through pain assessments (i.e., questionnaires like the Central Sensitization Inventory; performing quantitative sensory testing such as Conditioned Pain Modulation) [57, 58]. All these processes may provide useful information for clinicians, but they are not valid measurements of CS. These differences reveal the demand for more training and educational programs on pain.

The absence of standardized CS protocols and limited use of CS‐specific tools likely contribute to variability in diagnosis, delayed recognition of complex cases, and suboptimal tailoring of pharmacological and nonpharmacological interventions. In practice, this may translate into prolonged diagnostic odysseys, over‐reliance on stepwise analgesic escalation, and increased risk of pain chronification. Targeted educational programs and the integration of simple, time‐efficient CS screening tools into PC workflows for early and proper identification could help bridge this gap and promote earlier, more mechanism‐oriented management [21, 49, 59–61].

The present study reached a broad consensus on recommendations regarding the general therapeutic management of CNCP, following previous published guidelines and consensus [24, 26–28, 39]. It is important to identify the type of pain and the clinical sociodemographic characteristics of the patients to provide the most appropriate individualized treatment [24, 26–28, 62].

In particular, it is important to individually assess the patient before prescribing medications such as opioids, antidepressants, anticonvulsants, and antiseizure drugs. When considering elderly patients, the use of antipyretic analgesics, NSAIDs, and strong opioids was not recommended, reflecting caution from the panel [63, 64]. Indeed, antidepressants, anticonvulsants, and opioids are potentially sedating medications and involve the risk of confusion and falls, and clinicians should consider this when prescribing them to older adults. In addition, tricyclic antidepressants have an anticholinergic effect and are not recommended for older adults [65]. Therefore, prescriptions for the elderly should be made on a case‐by‐case basis [64]. Weak opioids were not recommended in patients undergoing chronic opioid treatment, because this drug combination may put the patient at risk for overdose [64]. For CNCP, drug combinations should be individualized considering patient needs, cost, convenience, comorbidities, risk‐benefit profile, etc. [64]. Apart from pharmacological treatment, nonpharmacological alternatives should be offered to patients; however, only 48% of patients are managed with a multicomponent approach, according to a patient survey [6]. Indeed, most patients report being dissatisfied with their treatment [6, 9]. This might be partially addressed by improving patients’ education on diagnosis and disease characteristics to best manage their expectations. Unfortunately, sometimes, it is the healthcare provider who does not have this information [6]. Again, it underscores the importance of a multidisciplinary approach and follow‐up, including nursing, psychology, physiotherapy, and pharmacy.

A wide consensus was reached on patient referral throughout the patient journey, in accordance with published referral recommendations [24, 26–28, 59, 66]. It is important to establish well‐defined referral criteria, although these are not always easy to apply in the real world because their application will also depend on available material and human resources. Nevertheless, good referral criteria constitute a well‐defined ultimate goal to be achieved, leading the way to optimal management. A good referral is important to avoid pain chronification and development of symptoms potentially associated with CS, as it provides optimal and timely treatment personalized to the patient’s profile and pain type [60].

The main strength of our study derives from the participation of a high number of panelists with different medical specialties from across Spain involved in CNCP diagnosis and management, thus ensuring representativeness. This panel provided valuable data on the aspects that should be reinforced to prioritize areas requiring particular attention. Importantly, it is the first Spanish consensus addressing CS, which is a term with terminology that is still unclear or not yet fully assimilated. Finally, strengths associated with the nature of the study design also have to be highlighted: (1) It provides knowledge and expertise from a wide panel of experts on CNCP. (2) Anonymity encourages honesty of responses by eliminating the influence of dominant figures and reducing group bias. (3) The design with two rounds allows refinement of the responses to get a higher consensus and more informed questions [67].

### 4.1. Limitations of the Study

Despite the strengths of the Delphi method, several inherent limitations of the Delphi design cannot be ignored, such as the lack of direct interaction with participants. It limits discussion and debate of points of view and may result in the loss of valuable information [68]; however, it is mitigated by the presence of the multidisciplinary SC. In this respect, including only physicians is a limitation that prevents generalization of the conclusions. Nevertheless, this aligns with the Delphi method to ensure that consensus is achieved by experts on the study subject [68].

A potential limitation identified during study planning was the risk of selection bias inherent to recruitment through the sponsor’s network. To mitigate this, participation was restricted to panelists who met predefined criteria of clinical expertise (years of experience and recognized professional trajectory) and the sample was designed to ensure broad geographic and practice‐setting representation across Spain. Additionally, the questionnaire did not include any items that could directly relate to or influence the sponsor’s activities or products, thereby reducing the likelihood of sponsor‐driven bias and strengthening the validity of the consensus obtained.

In this respect, another important limitation relates to the structure and length of the questionnaire. With 168 items to evaluate, participant fatigue could have influenced responses, potentially introducing bias due to exhaustion. While participants were allowed to respond partially and continue later, and received compensation to support engagement, fatigue remains a relevant concern. This issue is compounded using a 9‐point Likert scale, which can be susceptible to central tendency bias, where respondents avoid extreme categories to steer clear of appearing to hold extreme views. This bias is particularly problematic in lengthy questionnaires, as participants may “reserve” stronger responses for later items or simplify earlier answers, leading to uneven response patterns that cannot be fully corrected by standard normalization techniques.

Additionally, the inclusion of yes/no (dichotomous) questions, which were not part of the consensus process but intended to explore routine clinical practices, may have limited the granularity of information obtained from these items.

Furthermore, although pain is a subjective experience, the study did not include patient perspectives, which may limit the comprehensiveness of the findings. Finally, as this study was conducted within the Spanish healthcare context, caution is advised when extrapolating these findings to other geographical or healthcare settings.

## 5. Conclusion

This study provides physician‐based recommendations for the detection, management, and follow‐up of CS and CNCP within the Spanish clinical setting. Our findings underscore the need to recognize pain as an entity in its own right and to ensure its appropriate documentation in medical records. CNCP should be approached holistically and managed through multidisciplinary collaboration. In addition, the presence of pain should be proactively assessed in high‐risk groups, such as patients with mood disorders and older adults. The study also highlights the relevance of CS‐related symptoms in CNCP evolution and the current lack of consolidated knowledge on CS in routine practice. Addressing this knowledge gap is essential, as insufficient awareness and delayed recognition of CS‐related symptoms can hinder early intervention, contribute to pain chronification, and lead to inconsistent diagnostic and therapeutic decisions. Closing this gap is therefore critical to ensure timely, accurate assessment and to optimize long‐term outcomes in patients with CNCP. Finally, improving education, for both healthcare professionals and patients, remains essential to support optimal, patient‐centered care.

## Author Contributions

Carlos Tornero, Pedro Juan Ibor Vidal, Carlos Ibor Miguel, Manuel Maestre Moreno, and Luis Javier Roca Ruiz as SC contributed to the conceptualization and design of the Delphi survey, interpretation and validation of the data, and the final manuscript.

Angela Magrone Massari led the funding acquisition and contributed as support to methodology, project administration, resources, supervision, and medical writing (review and editing).

Maria Ruart contributed to the study design, data collection, analysis and interpretation, and medical writing.

## Funding

This study was funded by Grupo Ferrer Internacional, S.A.

## Disclosure

The present study has been funded by Ferrer Internacional, S.A., without influencing the design, implementation, or interpretation of the study’s results.

## Ethics Statement

This study is a Delphi expert consensus survey and does not constitute a clinical trial. The participants were healthcare professionals recognized as experts in the field who were asked to provide their opinions on specific issues related to chronic noncancer pain (CNCP) and central sensitization (CS). No patients participated in the study, and the experts did not undergo any interventions or the collection of biological samples. In accordance with the ISPOR Good Practices for Outcomes Research (2017), specifically Sections 9.2 and 9.3 from Appendix No. 9; and the EPHMRA Code of Conduct (2023), specifically Sections 1.2 and 1.3, as well as examples from similar published studies, ethical review is not required for this type of noninterventional study. Only experts who provided consent to participate through the online platform were included in the Delphi survey. Data were analyzed anonymously while ensuring confidentiality and privacy. Additionally, the content of the questionnaire does not pertain to clinical trials or medical scientific research involving patient data, nor does it compromise the physical or psychological integrity of any subject. The authors confirm that no artificial intelligence tools or technologies were used in the design or conduct of the study, nor in the writing or preparation of this manuscript.

## Conflicts of Interest

It is disclosed that the panelists who replied to the Delphi questionnaire and the members of the scientific committee who developed the questionnaire for this Delphi study have received a modest honorarium commensurate with the time required to complete both Delphi rounds, in line with usual practice for advisory and consensus activities in Spain. This compensation has not influenced the design, implementation, or interpretation of the study’s results. It was also independent of individual responses and final consensus, and was not contingent on agreement with the sponsor or the SC. The integrity and independence of the results have been ensured through a rigorous review and analysis process. Maria Ruart is a full‐time employee at Adelphi Targis S.L., and Angela Magrone is a full‐time employee at Ferrer Internacional, S.A. The authors declare that their professional activities or interests within these companies had no influence on the design, conduct, analysis, or interpretation of the results of the present study. The rest of the authors declare no conflicts of interest.

## Supporting Information

Additional supporting information can be found online in the Supporting Information section.

## Supporting information


**Supporting Information 1** Supporting Table 1: Statements on CNCP definition. Supporting 2. Supporting Table 2: Recommendations on detection and diagnosis of CNCP. Supporting 3. Supporting Table 3: Recommendations on CNCP treatment. Supporting 4. Supporting Table 4: Recommendations on CNCP follow‐up.

## Data Availability

The data that support the findings of this study are available from the corresponding author upon reasonable request.
