# Supplementary material for: Early and Multidisciplinary Management of Chronic Noncancer Pain and Conceptualization of Central Sensitization in Spain: Delphi CADOR Study
Source: Pain Res Manag. 2026 Oct 4;2026:1295794. doi: 10.1155/prm/1295794 (PMC13635690; doi:10.1155/prm/1295794)
Supplement: Supplementary file 1 [file PRM-2026-1295794-s001.docx]

**SUPPLEMENTARY TABLES**

**Supplementary Table 1: Statements on CNCP definition.**

| **Statement** | **Disagreement**  **(% of consensus)** | **Neutral**  **(% of consensus)** | **Agreement**  **(% of consensus)** | **Final consensus** |
| --- | --- | --- | --- | --- |
| **1)** CNCP is defined as pain present for more than three months, continuously or intermittently, more than five days per week. | 3% | 6% | **91%** | Agreement |
| **2)** CNCP is characterized by impaired functional capacity. | 2% | 12% | **86%** | Agreement |
| **3)** CNCP is a disease. | 2%  (7% in the 1^st^ round) | 14%  (26% in the 1^st^ round) | **84%**  (66% in the 1^st^ round) | Agreement* |
| **4)** The CNCP is characterized by moderate or high intensity on the visual analogue scale. | 3%  (7% in the 1^st^ round) | 17%  (27% in the 1^st^ round) | **80%**  (67% in the 1^st^ round) | Agreement* |

**Notes:** *Items that underwent a second round of voting.

**Abbreviations:** CNCP: Chronic Non-Cancer-Pain.

**Supplementary Table 2: Recommendations on detection and diagnosis of CNCP.**

| **Statement** | **Disagreement**  **(% of consensus)** | **Neutral**  **(% of consensus)** | **Agreement**  **(% of consensus)** | **Final consensus** |
| --- | --- | --- | --- | --- |
| **Degree of agreement that the CNCP should be recorded in the patient's medical record** | | | | |
| **1)** Indicate your degree of agreement whether CNCP should be recorded as a disease in its own right in the medical record, according to ICD-11 | 4% | 16% | **80%** | Agreement |
| **Importance of looking into the presence of pain in certain patient profiles** | | | | |
|  | **No (% of agreement)** |  | **Yes (% of agreement)** |  |
| **2)** Do you think you should ask about the presence of pain regardless of the reason for consultation in specific patient profiles? (Select patient profiles)^α^ | 11% | - | **89%** | Agreement* |
| **2a)** Mood disorders (anxiety, depression, catastrophizing, or other). |  |  | **74.2%** | Agreement* |
| **2b)** Age >70 years old^a^ |  |  | **73%** | Agreement* |
| **2c)**Surgeries |  |  | 67%  (48% in the 1^st^ round) | Undetermined* |
| **2d)** Age 50-70 years old |  |  | 52%  (64.6% in the 1^st^ round) | Undetermined* |
| **2e)** Women |  |  | 27%  (12% in the 1^st^ round) | Undetermined* |
| **2f)** Obesity |  |  | 25%  (32% in the 1^st^ round) | Undetermined* |
| **2g)** Adolescent |  |  | 11%  (54%% in the 1^st^ round) | Undetermined* |
| **Statement** | **Disagreement**  **(% of consensus)** | **Neutral**  **(% of consensus)** | **Agreement**  **(% of consensus)** | **Final consensus** |
| **Diagnosis of the CNCP** | | | | |
| **3)** Assessing the impact of pain on the patient's quality of life and functional capacity is essential in the diagnosis of CNCP. | 1% | 4% | **95%** | Agreement |
| **4)** Both surgical and non-surgical specialties require specific training for the detection and diagnosis of post-surgical CNCP. | 1% | 9% | **90%** | Agreement |
| **5)** Primary Care should play an essential role in detection/diagnosis as it is the first point of entry of the patient into the health care system. | 1% | 10% | **89%** | Agreement |
| **6)** The complexity of CNCP diagnosis lies in its classification and evaluation. | 1%  (6% in the 1^st^ round) | 17%  (27% in the 1^st^ round) | **82%**  (69% in the 1^st^ round) | Agreement* |
| **7)** The application of validated pain assessment scales/tools (intensity, type of pain, quality of life, etc.) is an essential element in the detection and diagnosis of CNCP. | 1% | 17% | **82%** | Agreement |
| **8)** The detection and possible suspicion of a diagnosis of CNCP should be made by the first healthcare professional the patient sees (regardless of the origin of the pain). | 2% | 24% | **74%** | Agreement |
| **Diagnostic criteria** | | | | |
| **9)** Pain that affects the quality of physical, psychological and social life. | 0% | 4% | **96%** | Agreement |
| **10)** Presence of pain lasting ≥ 3 months and intermittent pain ≥ 5 days a week. | 1% | 5% | **94%** | Agreement |
| **11)** Pain that persists beyond the expected time for tissue healing or resolution of the underlying disease. | 3% | 16% | **81%** | Agreement |
| **12)** Presence of pain of an intensity ≥3 on the Verbal Numerical Scale (VNS). | 10%  (7% in the 1^st^ round) | 32%  (41% in the 1^st^ round) | 58%  (52% in the 1^st^ round) | Undetermined* |
| **13)** The onset of pain is progressive^b^ | 7%  (18% in the 1^st^ round) | 36%  (49% in the 1^st^ round) | 57%  (33% in the 1^st^ round) | Undetermined* |
| **Evaluation in the initial characterization** | | | | |
| **14)** Location of pain (focal, multifocal, generalized, reflex, superficial or deep). | 0% | 3% | **97%** | Agreement |
| **15)** Impact on subjective functional capacity: daily life, work, leisure, sleep. | 0% | 4% | **96%** | Agreement |
| **16)** Previous treatments and response to them. | 0% | 4% | **96%** | Agreement |
| **17)** Influencing factors: aggravating and alleviating factors. | 0% | 4% | **96%** | Agreement |
| **18)** Chronology (morning-night, rest-motion, continuous-intermittent). | 0% | 6% | **94%** | Agreement |
| **19)** Pathophysiological origin: nociceptive, neuropathic, mixed. | 0% | 8% | **92%** | Agreement |
| **20)** Definition of pain: penetrating, stabbing, oppressive. | 0% | 10% | **90%** | Agreement |
| **21)** Screening for anxiety and depression. | 1% | 11% | **88%** | Agreement |
| **22)** Lifestyle habits (healthy diet and physical exercise) and toxins. | 0% | 12% | **88%** | Agreement |
| **23)** Psychosocial history (work, family situation...). | 0% | 12% | **88%** | Agreement |
| **24)** Motor system: strength, tone and musculoskeletal reflexes, palpation of pulses and auscultation of murmurs of the affected areas. | 2% | 13% | **85%** | Agreement |
| **25)** Pain map, somatosensory system exploration: tactile sensitivity, presence of allodynia or hyperalgesia (cotton and pin), and vibratory sensitivity (tuning fork). | 1% | 14% | **85%** | Agreement |
| **26)** Physical examination: weight, height, BMI, blood pressure, etc., and a systematized examination by apparatus. | 2% | 18% | **80%** | Agreement |
| **27)** Complementary tests: imaging techniques. | 5% | 21% | **74%** | Agreement |
| **Classification of pain-pathophysiological cause** | | | | |
| **28)** CNCP can be diagnosed, even if its pathophysiological origin is not known. | 1% | 16% | **83%** | Agreement |
| **29)** Knowledge of the pathophysiology of pain is essential for a correct diagnosis of the patient with CNCP. | 2% | 22% | **76%** | Agreement |
| **30)** Pain classification should be performed by Primary Care. | 4% | 24% | **72%** | Agreement |
| **General concepts on CS** | | | | |
| **31)** Sufficient resources and time should be provided to explain to the CS patient what his or her medical condition consists of and the therapeutic options available. | 0% | 4% | **96%** | Agreement |
| **32)** CS is defined as an amplification of nociceptive signals within the central nervous system, resulting in hypersensitivity to pain. | 0% | 6% | **94%** | Agreement |
| **33)** CS should be recognized as a medical condition with a negative impact on the quality of life of those who suffer from it. | 1% | 4% | **94%** | Agreement |
| **34)** The diagnosis of CS should be made by Primary Care with support from other clinical areas such as Rehabilitation, Traumatology, Rheumatology and/or Pain Unit. | 1% | 6% | **93%** | Agreement |
| **35)** CS should be considered in all patients, regardless of the type of pain. | 4% | 25% | **71%** | Agreement |
| **36)** Acute pain can cause CS, reducing the pain threshold and increasing the response to noxious stimuli. | 3% | 26% | **71%** | Agreement |
| **37)** The detection and possible diagnosis of CS should be performed by Primary Care. | 5%  (7% in the 1^st^ round) | 27%  (32% in the 1^st^ round) | 68%  (61% in the 1^st^ round) | Undetermined* |
| **Reasons for lack of knowledge about the impact of CS on pain** | | | | |
| **38)** In my usual care center, I do not have enough time during consultations for the evaluation and possible diagnosis of CS. | 0% | 19% | **81%** | Agreement |
| **39)** In my usual clinical practice, cases of complex patients and those with CS are treated by other specialties. | 13% | 13% | **74%** | Agreement |
| **40)** No protocols or strategies for the diagnosis and/or management of CS are available at my usual care center. | 6% | 19% | **74%** | Agreement |
| **41)** Not enough pain training courses are offered in my day-to-day life^c^ | 12%  (6% in the 1^st^ round) | 35%  (45% in the 1^st^ round) | 53%  (48% in the 1^st^ round) | Undetermined* |
| **Evaluation of CS-related symptoms** | | | | |
| **42)** Hyperalgesia and/or allodynia | 2% | 8% | **90%** | Agreement |
| **43)** The patient experiences pain in major activities of daily living. | 0% | 11% | **89%** | Agreement |
| **44)** The distribution of pain is diffuse and generalized. | 0% | 14% | **86%** | Agreement |
| **45)** No evidence of tissue damage or disease. | 3% | 13% | **84%** | Agreement |
| **46)** The presence of pain with moderate-severe intensity. | 2% | 18% | **80%** | Agreement |
| **47)** Presence of pain for more than 3 months. | 2% | 19% | **79%** | Agreement |
| **Referral criteria at the time of diagnosis** | | | | |
| **48)** In patients diagnosed with CNCP who present limitations in joint ranges, referral to a Rehabilitation specialist can be considered. | 2% | 8% | **90%** | Agreement |
| **49)** In the case of patients diagnosed with uncontrolled CNCP of musculoskeletal origin and/or with suspected surgical indication, referral to Traumatology may be considered. | 3% | 8% | **89%** | Agreement |
| **50)** Unbearable pain with a Verbal or Visual Analog Scale equal to or greater than 8. | 3% | 16% | **81%** | Agreement |
| **51)** In the case of fragile patients such as pregnant women or polymedicated patients, referral to Rehabilitation or Pain Unit for interventional techniques may be considered. | 3% | 18% | **79%** | Agreement |
| **52)** Suspected presence of CS. | 4% | 18% | **78%** | Agreement |
| **53)** Detection of painful diabetic neuropathy, postherpetic neuralgia, cervical and lumbar radiculopathy or entrapment neuropathies. | 7% | 16% | **77%** | Agreement |
| **54)** Polymedicated patients in whom intolerable adverse effects with medication or important interactions are present or may occur. | 3%  (7% in the 1^st^ round) | 21%  (24% in the 1^st^ round) | **76%**  (69% in the 1^st^ round) | Agreement* |
| **55)** Impossibility of performing diagnostic tests from Primary Care. | 15%  (18% in the 1^st^ round) | 15%  (16% in the 1^st^ round) | **70%**  (66% in the 1^st^ round) | Agreement* |

**Notes**: *Items that underwent a second round of voting. ^α^Question with multi-response format. ^a^In the first wave, this statement was formulated as follows: "Age > 50 years." After the first wave, the Scientific Committee (SC) decided to modify it to improve its comprehensibility in the second wave. ^b^In the first wave, this statement was formulated as follows: "The onset of pain is definite." After the first wave, the SC decided to modify it to improve its understanding in the second wave. ^c^In the first wave, this statement was worded as follows: "No pain training courses are offered in my usual clinical practice." After the first wave, the SC decided to modify it to improve its understanding in the second wave.

**Abbreviations**: BMI: Body Mass Index; CNCP: Chronic Non-Cancer Pain; CS: Central Sensitization; ICD-11: International Classification of Diseases 11^th^ revision.

**Supplementary Table 3:** **Recommendations on CNCP treatment.**

| **Statement** | **Disagreement**  **(% of consensus)** | **Neutral**  **(% of consensus)** | **Agreement**  **(% of consensus)** | **Final consensus** |
| --- | --- | --- | --- | --- |
| **Importance of the different therapeutic targets in patients with CNCP**^†^ | | | | |
| **1)** Reduce the intensity of pain (reduction of the VNS level ≥ 30% of the initial value or of the BPI-SF of 2 points) and reduce its functional repercussion. | 12% | 3% | **85%** | Agreement |
| **2)** Preservation of work, social and family activities. | 40%  (34% in the 1^st^ round) | 22%  (22% in the 1^st^ round) | 38%  (40% in the 1^st^ round) | Undetermined |
| **3)** Preserving the sleep pattern. | 42%  (53% in the 1^st^ round) | 26%  (26% in the 1^st^ round) | 32%  (21% in the 1^st^ round) | Undetermined |
| **4)** Maintaining or improving healthy lifestyle habits and physical activity. | 57%  (55% in the 1^st^ round) | 16%  (16% in the 1^st^ round) | 27%  (29% in the 1^st^ round) | Undetermined |
| **5)** Improve mood. | 49%  (42% in the 1^st^ round) | 33%  (33% in the 1^st^ round) | 18%  (25% in the 1^st^ round) | Undetermined |
| **General handling CNCP** | | | | |
| **6)** Communication between the physician and the patient should be improved so as not to create false expectations regarding the efficacy of the treatment. | 0% | 6% | **94%** | Agreement |
| **7)** Regardless of the pathophysiological cause, special treatment and care is required in chronic pain early on. | 0% | 6% | **94%** | Agreement |
| **8)** It is important to assess adherence to treatment for both underutilization and overutilization. | 0% | 7% | **93%** | Agreement |
| **9)** Non-pharmacological alternatives should be offered to patients with CNCP, such as the implementation of healthy habits like physical exercise, physiotherapy, psychological, etc. | 0% | 8% | **92%** | Agreement |
| **10)** Educating the CS patient about his or her medical condition can improve adherence to treatment. | 0% | 10% | **90%** | Agreement |
| **11)** Opioids are sometimes indicated for CNCP. | 0% | 10% | **90%** | Agreement |
| **12)** Minimally invasive procedures should be considered from the outset, even in mild to moderate pain intensity, as they can abort the temporary course of pain and prevent its chronification and CS phenomena. | 1% | 11% | **88%** | Agreement |
| **13)** Inadequate treatment leads to chronification of pain. | 1% | 11% | **88%** | Agreement |
| **14)** Patients with CNCP and pathology of the locomotor system would benefit from treatment by means of physical therapy, both electrotherapy and physical exercise prescribed by Primary Care or other specialties. | 1% | 12% | **87%** | Agreement |
| **Degree of agreement regarding the concern of CS associated symptoms in the treatment and prognosis of patients with CNCP** | | | | |
| **15)** Please indicate your degree of agreement regarding your concern about CS regarding the treatment and prognosis of patients with CNCP, with 9 being the highest concern and 1 being the lowest concern. ^‡^ | 1% | 17% | **82%** | Agreement |
| **Degree of agreement regarding the concern of CS associated symptoms in the treatment and prognosis of patients with CNCP** | | | | |
| **16)** The clinical areas that should be involved in the pharmacological treatment of patients with CS range from Primary Care to Rehabilitation, Traumatology, Rheumatology and/or Pain Unit. | 0% | 6% | **94%** | Agreement |
| **17)** Any type of pain according to its pathophysiology should be treated by Primary Care and other clinical areas such as Rehabilitation, Traumatology, Rheumatology and/or Pain Unit in complex cases (patients who do not respond to the treatment of the corresponding step). | 1% | 6% | **93%** | Agreement |
| **18)** Mixed CNCP poses an added difficulty in patient management and should be treated by a multidisciplinary team. | 1% | 7% | **92%** | Agreement |
| **Use of antipyretic analgesics and non-steroidal anti-inflammatory drugs** | | | | |
| **Statement** | **No (% of agreement)** |  | **Yes (% of agreement)** | **Final consensus** |
| **19)** For mild pain | 9% |  | **91%** | Agreement |
| **20)** Use of this treatment as a first step | 11% |  | **89%** | Agreement |
| **21)** In young patients under 18 years of age | 14% |  | **86%** | Agreement |
| **22)** For moderate pain | 23% |  | **77%** | Agreement |
| **23)** As a treatment for nociceptive pain | 27% |  | **73%** | Agreement |
| **24)** Patients with comorbidities^§^ | **88%** |  | 12% | Disagreement |
| **25)** As a treatment for neuropathic pain | **86%** |  | 14% | Disagreement |
| **26)** Use of this treatment as a third step | **84%** |  | 16% | Disagreement |
| **27)** As a treatment for CS | **75%** |  | 25% | Disagreement |
| **28)** For severe pain | **74%** |  | 26% | Disagreement |
| **29)** Use of this treatment as a second step | **73%** |  | 27% | Disagreement* |
| **30)** Polymedicated patients | **70%** |  | 30% | Disagreement* |
| **31)** Patients with suboptimal pain control/no pain control | 69%  (64% in the 1^st^ round) |  | 31%  (27% in the 1^st^ round) | Undetermined* |
| **32)** In elderly patients | 59%  (57% in the 1^st^ round) |  | 41%  (43% in the 1^st^ round) | Undetermined* |
| **33)** Patient in chronic treatment with opioids | 53%  (55% in the 1^st^ round) |  | 47%  (45% in the 1^st^ round) | Undetermined* |
| **Use of weak opioids** | | | | |
| **34)** As treatment of nociceptive pain | 10% |  | **90%** | Agreement |
| **35)** Patients with comorbidities^§^ | 11% |  | **89%** | Agreement |
| **36)** In elderly patients | 11% |  | **89%** | Agreement |
| **37)** For moderate pain | 14% |  | **86%** | Agreement |
| **38)** Patients with suboptimal pain control/no pain control | 16% |  | **84%** | Agreement |
| **39)** As a treatment for CS | 17% |  | **83%** | Agreement |
| **40)** Use of this treatment as a second step | 18% |  | **82%** | Agreement |
| **41)** Polymedicated patients | 21% |  | **79%** | Agreement |
| **42)** As a treatment for neuropathic pain | 27% |  | **73%** | Agreement |
| **43)** For severe pain | 27% |  | **73%** | Agreement |
| **44)** For mild pain | **90%** |  | 10% | Disagreement |
| **45)** Use of this treatment as a first step | **85%** |  | 15% | Disagreement |
| **46)** In young patients under 18 years of age | **72%** |  | 28% | Disagreement* |
| **47)** Use of this treatment as a third step | 44%  (4% in the 1^st^ round) |  | 56%  (57% in the 1^st^ round) | Undetermined* |
| **48)** Patient in chronic treatment with opioids | 68%  (52% in the 1^st^ round) |  | 32%  (48% in the 1^st^ round) | Undetermined* |
| **Use of strong opioids** | | | | |
| **49)** For severe pain | 4% |  | **96%** | Agreement |
| **50)** Use of this treatment as a third step | 5% |  | **95%** | Agreement |
| **51)** Patients with suboptimal pain control/no pain control | 9% |  | **91%** | Agreement |
| **52)** As a treatment for nociceptive pain | 18% |  | **82%** | Agreement |
| **53)** As a treatment for CS | 20% |  | **80%** | Agreement |
| **54)** As a treatment for neuropathic pain | 20% |  | **80%** | Agreement |
| **55)** Polymedicated patients | 21% |  | **79%** | Agreement* |
| **56)** Patients with comorbidities^§^ | 24% |  | **76%** | Agreement* |
| **57)** Patient in chronic treatment with opioids. | 30% |  | **70%** | Agreement |
| **58)** For mild pain | **95%** |  | 5% | Disagreement |
| **59)** Use of this treatment as a first step | **93%** |  | 7% | Disagreement |
| **60)** Use of this treatment as a second step | **82%** |  | 18% | Disagreement |
| **61)** For moderate pain | **79%** |  | 21% | Disagreement |
| **62)** In young patients under 18 years of age | **76%** |  | 24% | Disagreement |
| **63)** In elderly patients | 46%  (50% in the 1^st^ round) |  | 54%  (50% in the 1^st^ round) | Undetermined* |
| **Use of antidepressants, antiseizure or antiepileptic drugs** | | | | |
| **64)** As a treatment for neuropathic pain | 3% |  | **97%** | Agreement |
| **65)** As a treatment for CS | 7% |  | **93%** | Agreement |
| **66)** Patients with suboptimal pain control/no pain control | 8% |  | **92%** | Agreement |
| **67)** For severe pain | 9% |  | **91%** | Agreement |
| **68)** Patient on chronic opioid treatment | 10% |  | **90%** | Agreement |
| **69)** In elderly patients | 19% |  | **81%** | Agreement |
| **70)** Polymedicated patients | 20%  (39% in the 1^st^ round) |  | **80%**  (61% in the 1^st^ round) | Agreement* |
| **71)** For moderate pain | 20%  (35% in the 1^st^ round) |  | **80%**  (67% in the 1^st^ round) | Agreement* |
| **72)** Use of this treatment as a third step | 23% |  | **77%** | Agreement |
| **73)** As treatment of nociceptive pain | 25%  (49% in the 1^st^ round) |  | **75%**  (51% in the 1^st^ round) | Agreement* |
| **74)** Patients with comorbidities^§^ | 28% |  | **72%** | Agreement |
| **75)** Use of this treatment as a second step | 28%  (46% in the 1^st^ round) |  | **72%**  (54% in the 1^st^ round) | Agreement* |
| **76)** For mild pain | **90%** |  | 10% | Disagreement |
| **77)** In young patients under 18 years of age | **84%** |  | 16% | Disagreement* |
| **78)** Use of this treatment as a first step | **83%** |  | 17% | Disagreement |
| **Referral criteria at the time of treatment** | | | | |
| **79)** Patients with severe pain, who have not responded to pharmacological treatment. | 1% | 9% | **90%** | Agreement |
| **80)** Patients with very high doses of oral opioids when doses equivalent to 90-120 mg of morphine are exceeded. | 2% | 8% | **90%** | Agreement |
| **81)** Those patients/pathologies that, as a consequence of CNCP, present restrictions in the range of mobility should be referred to the Rehabilitation service. | 2% | 12% | **86%** | Agreement |
| **82)** Patients with allergy to a broad spectrum of drugs (including analgesics), intolerance or side effects. | 2% | 15% | **83%** | Agreement |
| **83)** In patients with a diagnosis of CNCP of osteoarticular origin who undergo physical treatment without clinical improvement, with or without analgesic medication, referral to the Rehabilitation Service may be considered. | 6% | 13% | **81%** | Agreement |
| **84)** Patient who already has an established chronic degenerative disease, acute clinical manifestations, different from the underlying disease, less than 3 months old, which invalidates him/her and limits his/her basic autonomy. | 4% | 15% | **81%** | Agreement |
| **85)** Patients with a medical condition for which the use of certain drugs for the treatment of pain is contraindicated. | 3% | 16% | **81%** | Agreement |
| **86)** Loss of oral route for the administration of analgesic drugs in patients where the instability of the process does not recommend the transdermal route and their life expectancy does not recommend the subcutaneous route. | 5% | 20% | **75%** | Agreement |

**Notes**: *Items that underwent a second round of voting. ^†^This question was considered as a consensus-type question itself. The level of importance for each therapeutic objective had to be ranked from 1 (most important) to 5 (least important). It was considered that the panelists reached consensus on what should be the main therapeutic objective among the 5 proposed. ^‡^Specifically for this item, the degree of consensus was determined as follows: Little concern (1-3), Neutral (4-6), Great concern (7-9). ^§^Renal failure, liver failure, cardiac or respiratory failure, arterial hypertension, cerebral and cerebrovascular diseases, digestive diseases (eg peptic ulcers), obesity, anxiety.

**Abbreviations**: BPI-SF: Brief Pain Inventory-Short Form; CNCP: Chronic Non-Cancer Pain; CS: Central Sensitization; VNS: Verbal Numerical Scale.

**Supplementary Table 4:** **Recommendations on CNCP follow-up.**

| **Statement** | **Disagreement**  **(% of consensus)** | **Neutral**  **(% of consensus)** | **Agreement**  **(% of consensus)** | **Final consensus** |
| --- | --- | --- | --- | --- |
| **General Concepts** | | | | |
| **1)** Follow-up should be closer in case of patients with comorbidities^§^ | 1% | 5% | **94%** | Agreement |
| **2)** It should be taken into account that the follow-up time criteria should be personalized according to the characteristics of the patient and the means available to the health professional. | 1% | 5% | **94%** | Agreement |
| **3)** Patients who start treatment with opioids should be closely monitored for possible adverse effects and the efficacy of the treatment should be evaluated after 15 days. | 0% | 10% | **90%** | Agreement |
| **4)** Follow-up should be performed by Primary Care in a controlled patient. | 2% | 8% | **90%** | Agreement |
| **5)** Follow-up should be carried out by clinical areas such as Rehabilitation, Traumatology, Rheumatology and/or Pain Unit in complex cases (patients who do not respond to the treatment of the corresponding step). | 0% | 11% | **89%** | Agreement |
| **6)** The patient should be informed of the need for a multidisciplinary approach and follow-up in Primary Care, and this should be recorded in the Health History. | 0% | 13% | **87%** | Agreement |
| **7)** Patient follow-up should evaluate the same aspects as in the initial patient assessment (location, quality, intensity, characteristics or impact of pain) and the response to treatment (type, dose, duration, side effects) or reasons for discontinuation of treatment. | 1% | 14% | **85%** | Agreement |
| **8)** Telemedicine offers a new horizon in monitoring for all patients (with or without an outside residence); especially for the detection of adverse effects and to increase the patient's sense of security. | 6% | 10% | **84%** | Agreement |
| **9)** Nursing should have a role in the follow-up of patients with CNCP through outcome assessment, use of scales, adherence to treatment and/or canister review (duplicities). | 3% | 13% | **84%** | Agreement |
| **10)** Regardless of the type of pain and severity, the patient with CNCP should be referred to a patient education program throughout his or her treatment. | 4% | 18% | **78%** | Agreement |
| **11)** Patients with CNCP being treated with a pain relief drug should be evaluated generally every month and, depending on the evolution, periodically once a quarter. | 4% | 23% | **73%** | Agreement |
| **Referral criteria at follow-up** | | | | |
| **12)** Patients with CS whose pharmacological treatment is not effective. | 0% | 7% | **93%** | Agreement |
| **13)** Neuropathic pain not controlled with adequate pharmacological treatment adapted to the level and type of pain and the need for other techniques. | 1% | 7% | **92%** | Agreement |
| **14)** Patient not controlled with the third step. | 2% | 6% | **92%** | Agreement |
| **15)** Patients with chronic pain refractory to an adequate therapeutic scheme. | 1% | 8% | **91%** | Agreement |
| **16)** If at 6 months of treatment the pain intensity and/or functional goals are not achieved. | 1% | 9% | **90%** | Agreement |
| **17)** Detection of abuse in the case of major opioids, in case it is not possible to manage them. | 1% | 13% | **86%** | Agreement |
| **18)** Patients with compromised psychological evaluation. | 4% | 16% | **80%** | Agreement |
| **19)** Oral analgesic doses of opioids cause intolerable side effects, even if they are not excessively high. | 4% | 22% | **74%** | Agreement |
| **20)** Patients who present rapid tolerance to oral opioids. | 5% | 24% | **71%** | Agreement |

**Notes**: ^§^Renal failure, liver failure, cardiac or respiratory failure, arterial hypertension, cerebral and cerebrovascular diseases, digestive diseases (eg peptic ulcers), obesity, anxiety.

**Abbreviations:** CNCP: Chronic Non-Cancer Pain; CS: Central Sensitization.

**Supplementary Table 5:** **Dichotomous questions included in the questionnaire to gain further insights on clinicians’ clinical practice (not included in the consensus analysis).**

| **Questionnaire section** | **Question** | **Results** |
| --- | --- | --- |
| **CNCP detection and diagnosis** | *Please indicate whether you can record CNCP as a disease in patients' medical records according to the International Classification of Diseases (ICD-11) code.* | Yes 41.0%  **No 59.0%** |
|  | *Are you familiar with the impact of Central Sensitization (CS) on the pathophysiology of pain?* | **Yes 69.0%**  No 31.0% |
|  | *In your usual clinical practice, do you use any tools for detecting CS in patients with CNCP?* | Yes 12.0%  **No 51.0%**  Do not know which tools are available 37.0% |

**Abbreviations:** CNCP: Chronic Non-Cancer Pain; CS: Central Sensitization
